# Using quality assessment tools to critically appraise ageing research: a guide for clinicians

**DOI:** 10.1093/ageing/afw223

**Published:** 2016-12-08

**Authors:** Jennifer Kirsty Harrison, James Reid, Terry J Quinn, Susan Deborah Shenkin

**Affiliations:** 1 University of Edinburgh,Centre for Cognitive Ageing and Cognitive Epidemiology & The Alzheimer Scotland Dementia Research Centre , Edinburgh, United Kingdom; 2 Queen Elizabeth University Hospital, Medicine for the Elderly , Glasgow, United Kingdom; 3 University of Glasgow, Institute of Cardiovascular and Medical Sciences , Glasgow, United Kingdom; 4 University of Edinburgh, Geriatric Medicine, Department of Clinical and Surgical Sciences , Edinburgh, United Kingdom; 5 University of Edinburgh, Centre for Cognitive Ageing and Cognitive Epidemiology , Edinburgh, United Kingdom

**Keywords:** quality, assessment, methodology, reporting, critical appraisal, older people

## Abstract

Evidence based medicine tells us that we should not accept published research at face value. Even research from established teams published in the highest impact journals can have methodological flaws, biases and limited generalisability. The critical appraisal of research studies can seem daunting, but tools are available to make the process easier for the non-specialist. Understanding the language and process of quality assessment is essential when considering or conducting research, and is also valuable for all clinicians who use published research to inform their clinical practice.

We present a review written specifically for the practising geriatrician. This considers how quality is defined in relation to the methodological conduct and reporting of research. Having established why quality assessment is important, we present and critique tools which are available to standardise quality assessment. We consider five study designs: RCTs, non-randomised studies, observational studies, systematic reviews and diagnostic test accuracy studies. Quality assessment for each of these study designs is illustrated with an example of published cognitive research. The practical applications of the tools are highlighted, with guidance on their strengths and limitations. We signpost educational resources and offer specific advice for use of these tools.

We hope that all geriatricians become comfortable with critical appraisal of published research and that use of the tools described in this review – along with awareness of their strengths and limitations – become a part of teaching, journal clubs and practice.

## Background

Risk of bias, allocation and randomisation describe fundamental aspects of clinical research, but they are only useful if you know what they mean and how to apply them. Understanding how to assess published research quality is an essential skill for any clinician. For geriatricians, an ability to evaluate the available evidence and apply the findings to our population is essential. In this review we describe the importance of quality assessment; outline some assessment tools and reporting guidelines, and illustrate, with examples from published cognitive research studies, how the use of these tools can improve the quality of research and maximise its relevance to practice. We also highlight specific aspects of study design and conduct which can help support the inclusion of older people in research and maximise its value.

## What is ‘quality’ in relation to research?

Quality in research includes two complementary aspects: methodological and reporting quality. We consider both of these aspects in turn. Here ’quality’ does not relate to the importance of the topic or the enjoyability of the prose. The term is not universally popular, as some erroneously interpret ‘quality’ as relating to the value of the research. For brevity, and in keeping with current literature, we will use the term in this paper. Although relevant to all branches of medicine we highlight quality considerations for research relevant to geriatricians (see Supplementary data, Table S1 Appendix 2, available at *Age and Ageing* online).

## Methodological quality

Methodological quality relates to the design and conduct of research, and is fundamental to understanding the results and the level of confidence in the findings. We consider the important aspects of quality of conduct for each type of study.

## Randomised controlled trials

A well-conducted randomised controlled trial (RCT) is considered the highest form of evidence for assessing the effect of interventions, e.g. drug treatments [1]. In a RCT, one group is allocated to receive an intervention and the other receive a control. An example RCT compared the cognitive and functional outcomes between groups given Donepezil or placebo [**2**].

### Assessing bias in trials

Establishing the methodological quality of individual RCTs is largely based around assessment of the risk of bias. Bias can be defined as: ‘a systematic error, or deviation from the truth, in results or inferences’ [**3**]. Recognising the importance of a standardised approach to describing potential bias, Cochrane developed a tool to assess risk of bias in RCTs [**4**]. This considers bias in terms of *selection*, *performance*, *detection*, *attrition*, *reporting* and *other biases* and the user rates studies as high, low or unclear risk of bias for each domain [**4**]: these are described in detail, using the example of a RCT of donepezil and placebo in Table 1.

**Table 1.** Cochrane Risk of Bias assessment – with examples based around a randomised trial of Donepezil [**2**].

Form of bias & components	Explanation	Suggested best practice
Selection bias Random sequence generation Allocation concealment	How was the sequence for determining who would be in each group determined and how was the underlying method concealed	Centralised electronic method, separate from the research team so they cannot predict who will be allocated to which group and so cannot influence those receiving a preventive intervention
Performance bias Blinding of participants and personnel	How research participants or their clinical care team behave as a result of being in a trial.	Participants and their care team should not be aware if they are in the intervention or control group. In drug trials such as this a placebo medication is given to those in the control group so that individuals are not aware if they are receiving Donepezil or not.
Detection bias Blinding of outcome assessment	How was the outcome of interest assessed and who performed the assessment	Method for assessing the outcome (e.g. cognition, functional performance etc.) should be clearly stated, using validated tools where appropriate.Outcome assessment should be conducted by someone who was unaware of whether people were in the treatment or intervention group, ideally by an independent expert.
Attrition bias Incomplete outcome data	Is it clear how participants in the study moved through the stages of eligibility, recruitment, allocation and follow-up? Have individuals been lost, dropped-out of the study or died and, if so, are the reasons for this clear and reasonable?	Ideally this should be reported using a CONSORT flow diagram so the reader can judge the process themselves. It is important to evaluate if losses are unevenly balanced between groups, as this may reflect important effects of the intervention which limit the generalisability to the wider population (e.g. medication side effects/intolerance etc.)
Reporting bias Selective reporting	Have the authors reported the outcomes included in their study protocol.	A comprehensive study protocol is registered and accessible to the public, e.g. on clinicaltrials.gov. Reporting bias can occur when trial authors present additional unplanned analyses which show favourable results (e.g. on subgroups such as those with higher or lower MMSE scores etc.), or do not present planned analyses. Post-hoc analyses must be interpreted with caution as often the trial wasn't powered to look for these differences.
Other bias	Is the result likely to be generalisable to my population? This includes: – Baseline imbalances between groups – Different approaches to diagnosis from the intervention or control – Fraud	Successful randomisation should distribute characteristics evenly between groups, but by chance this may not be effective (e.g. co-morbidities such as depression may affect cognitive function if this is more common in one group than the other).

Considering ‘*performance bias’*, ideally participants should not be aware which arm of a study (intervention or control) they are part of. This can be problematic for complex interventions such as falls prevention, as it can be difficult to blind patients who are receiving specialist care. This often necessitates an assessment score of ‘high risk of bias’ when applying the tool. However, the creating a complex ‘sham intervention’ for the control group can have its own limitations. Logan *et al*. describe the unintended effect of providing an activity diary to their control arm of an occupational therapy intervention study for stroke survivors as minimising the true difference between the groups, as it stimulated activity among controls [**5**].

The category of ‘*other bias*’ is relevant in determining whether an RCT is valuable to practice by assessing whether the included participants are representative of your population. There is a recognition that many clinical trials have excluded older people, despite the intervention being studied being relevant to their needs [6]. Study design and conduct can take account of the needs of this population to increase their participation and thus the value of such trials. Risk of bias across a group of RCTs can be summarised graphically, generated by using the freely available ‘Review Manager’ software (see Supplementary data, Appendix 2, available at *Age and Ageing* online) [**7**].

Alternative approaches include scales – such as the Jadad scale [8] – which are designed to give a summary ‘quality’ score. These can lead to an over-simplification of quality assessment and are at greater risk of problems with inter-rater reliability, i.e. how different assessors respond to the same questions and how consistently they score the responses [9]. These scales have been criticised as, they can result in a RCT with a single significant flaw being considered as high quality, while a RCT with a number of minor biases would be classed as low quality. The use of quality scores in meta-analyses of clinical trials should therefore be avoided [**10**].

## Non-randomised and observational studies

Non-randomised and observational studies are not interchangeable terms. A non-randomised interventional study may compare the effects of an intervention (allocated in a non-random way) with a control [11]. Observational studies include cohort and case–control designs, and can all be described as non-randomised. There are a variety of quality tools for these studies, each with different indications and applications, and with no consensus agreement on a preferred tool [**12**,13]. Three tools are compared in see Supplementary data, Appendix 2, Table S3, available at *Age and Ageing* online.

Non-randomised studies where an intervention is being evaluated are primarily conducted where randomisation is not considered to be pragmatic or possible. One example is the Hospital Elder Life Programme, developed in the USA as a multicomponent programme to prevent delirium [**14**]. Here the authors wished to study their multicomponent intervention in one unit, compared with two control units, but they felt that they could not feasibly randomise participants to these units due to the large numbers requiring care in hospital [**14**].

Risk of bias is a key quality concern in non-randomised studies: systematic biases may influence the outcome in non-randomised studies, and this can be inflated in studies with large sample sizes, whereas in RCTs randomisation should minimise differences between the groups, and this is more effective the larger the study. For example, characteristics which might affect the incidence of delirium, such as presence of dementia, illness severity, etc. may not be equally distributed between the groups, potentially increasing the rates of delirium in one group compared to the other unrelated to the intervention. The Risk of Bias Assessment tool for Non-Randomised Studies (RoBANS) is a six-domain tool designed to look at risk of bias alone (Table 3) [**15**]. RoBANS is consistent with the Cochrane Risk of Bias tool [**4**] and thus simple diagrams can be created to transparently report the assessment of each study. It has been validated by its original authors, but has not yet had an external validation [**15**].

Observational studies (cohort and case–control designs) are used where randomised trials are not feasible or ethical or to provide initial evidence for interventions which can later be tested in randomised trials. Cohort studies prospectively follow groups who differ in their exposure to see how this exposure relates to the development of an outcome [16]. Case–control studies retrospectively compare individuals with a disease (‘cases’) with matched ‘controls’ without that disease, to understand which factors are associated with the development of the disease [16]. An example is whether delirium is associated with development of dementia [**17**]. It would be unethical, and perhaps impossible, to randomise individuals to experience delirium, therefore a cohort study follows individuals with and without delirium to determine who later develops dementia. The parameters under investigation are not controlled by the study design, and quality assessment, particularly the role of bias, is crucial.

Observational studies can provide evidence of associations between factors, but they cannot prove that an observed relationship is causative. The summary statistics of the strength of association can be adjusted for ‘potential confounders’ if data have been collected, such as age, sex, disease severity [**17**] to check that the association which has been observed persists after these have been accounted for. For example, if an increased likelihood of developing dementia after delirium is due to age differences between the groups. However, there is always the risk of ‘residual confounding’ – that results are due to variables that were not included in the analyses – and future RCTs may contradict the findings from observational studies.

The Newcastle-Ottawa Quality Assessment Scale (NOS) is a freely available eight-item scale with a version for cohort and case–control studies (Table 3) [**18**]. It has not yet been published in any peer-reviewed publication. The scale evaluates the domains of selection, comparability and outcome or exposure. One star is allocated when a feature of quality is present, to a maximum of nine (the comparability domain can score up to two stars) [**18**]. No specific value is assigned to high or low quality, although higher scores indicate greater use of favourable methodological aspects. The inter-rater reliability of the NOS has been questioned [**19**] as has its usefulness in distinguishing between studies of high and low quality [19, 20] and its lack of external validation makes it difficult to assess this.

## Diagnostic test accuracy studies

Diagnostic test accuracy studies evaluate the usefulness of a screening or diagnostic test(s) by comparing the performance of the test(s) against an established reference standard diagnostic approach [**21**]. The dementia diagnostic pathway requires diagnostic test accuracy research as a range of imaging tests, biomarkers, cognitive tests and informant instruments have been advocated [**22**]. A recent example featured evaluation of an informant instrument, the AD-8: the Washington University Dementia Screening Test, to diagnose cognitive impairment [**23**].

### Quality assessment in test accuracy

The Quality Assessment of Diagnostic Accuracy Studies-2 (QUADAS-2) tool is organised into four domains (*patient selection, index test, reference standard* and *flow and timing*) and, within these there are assessments of the *risk of bias* and, the *applicability of findings* [**24**]. Assessments are made for each domain, determining yes/no/unclear for each.

In dementia test accuracy:

- *Patient selection* considers the type of people included (e.g. cognitively intact, cognitively impaired), setting of recruitment, exclusion criteria (particularly exclusion of significant co-morbidities).
- Assessing *applicability* depends on judging how similar they are to the population seen in practice.
- The *index test* is whichever biomarker, imaging test, cognitive or informant tool being used while the *reference standard* is, for example, an expert clinical diagnosis.
  - The index test questions ask you to judge if the test was conducted as you would expect in practice, if it was independent of the reference standard and if any cut-off value was pre-specified or if these were decided based on the results.
  - The reference standard questions establish if the assessment was independent from the index test result and if the standard used is likely to correctly identify the condition, e.g. recognised diagnostic standard such as the International Classification of Disease (ICD) criteria.
- *Flow and timing* relates to the timing of administration of the test within the diagnostic encounter and whether all those participating in the study are clearly accounted for, to ensure there have been no exclusions which might mean the tool was less useful in practice (e.g. exclusion of those who have no carer/informant).

QUADAS-2 includes ‘signalling questions’ to help direct the interpretation of the tool questions [**24**]. Some test accuracy studies are at risk of incorporation bias as often, particularly for cognitive and informant tests, these inform the clinical expert diagnosis, thus studies can be penalised if these results have formed part of the reference standard. Graphical summary tables can be produced [**24**]. These quality assessment concepts are exemplified in a series of Cochrane reviews, including use of the Informant Questionnaire for Cognitive Decline in the Elderly (IQCODE) in secondary care [25].

## Systematic reviews and meta-analyses

Systematic reviews are the results of an organised synthesis of the evidence on a particular topic. Meta-analyses are a subgroup of systematic review where the same outcome measure is reported in multiple studies and the results can be combined statistically to calculate the estimated overall effect size from all the trials together. A recent Cochrane review sought to establish the evidence for the effectiveness of case management approaches to home support for people with dementia [**26**]. The outcomes which the authors wanted to describe included institutionalisation, hospital admission, mortality, quality of life, carer burden and service use [**26**]. Meta-analysis was possible for the outcome of institutional care, as these results were reported in eight studies at four different time points [**26**].

Well-conducted systematic reviews are valuable resources for researchers and clinicians, identifying where sufficient evidence exists for or against an intervention and where evidence is lacking. They not only summarise the available literature, but critically appraise it and summarise the confidence with which the conclusions are justified. If you are planning to undertake a systematic review it is advisable to consult your local librarian or information specialist who can provide support and expertise to help maximise the value of your work.

### Assessing the quality of a systematic review

The Assessing the Methodological Quality of Systematic Reviews (AMSTAR) tool [**27**] scores out of a total of 11 points. Key aspects include an *a priori* design, search method, quality assessment and how quality informs the review conclusions. There is no score which separates a high from a low quality review. AMSTAR has been validated by its original authors and has good inter-rater reliability and other favourable assessment score qualities [28, 29]. Similar reliability has been obtained in external validation [30].

### Assessing study quality when conducting a systematic review

It is necessary to assess the quality of the included studies in the review, using tools specific to the study design. If a systematic review is well conducted and robust, it may be possible to make recommendations about the quality of the identified evidence. This enables quantitative findings to be qualified with assessment of the quality. For example, if there are several studies of lower quality which are combined with one high quality RCT, the numerical results alone could lead to erroneous conclusions to be drawn if quality is not considered.

The Grading of Recommendations Assessment Development and Evaluation (GRADE) system was developed to improve the evaluation of evidence for clinical guidelines [**31**], and has been widely adopted by Cochrane, the World Health Organization and other healthcare organisations and journals [**32**]. It includes a *risk of bias* assessment, but also requires the assessment of *consistency, indirectness, imprecision* and *publication bias* [**31**].

- *Consistency* indicates whether the findings are consistent with those in the other included studies
- *Indirectness* indicates whether the evidence is applicable to the entire population or whether it is indirect and only applicable to a sub-set, e.g. exclusion of those with other significant co-morbidities such as depression would lead you to conclude a dementia research study was at risk of indirectness.
- *Imprecision* indicates the confidence interval associated with a finding, this will largely be reduced by studies with larger sample sizes, although not if the studies produce conflicting results.
- *Publication bias* can occur if not all studies conducted in the field are published and available for inclusion. Typically this occurs when studies with a negative result are not published, leading to a summary estimate of positive results which may be biased in this direction. This is more common in studies of interventions and can only be formally assessed where data are available for direct comparisons between studies.

**Figure 1.**
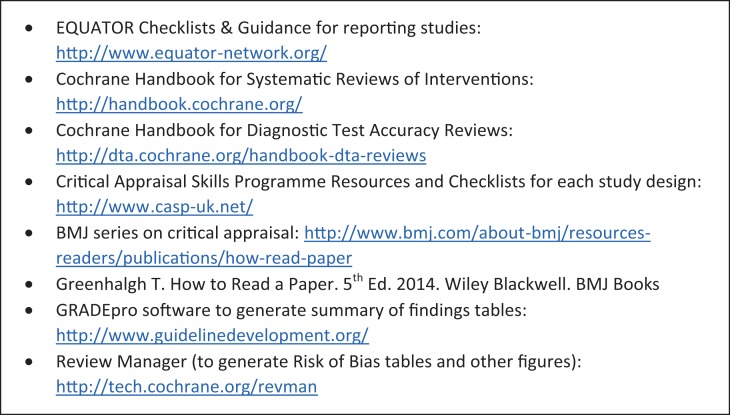
Useful links to resources for quality assessment and reporting of studies.

It is possible to assess the quality of each finding from a systematic review by applying GRADE, allowing you to present the statistical findings from each result alongside an assessment of the quality of the evidence from which the result is derived, transparently accounting for why evidence has been downgraded. Its use is not limited to RCTs, but many of the parameters can only be assessed using quantitative data and downgrading is required for study designs other than RCTs. As a system GRADE lends itself well to identifying areas for which further research evidence is required to improve the quality of the evidence. The GRADEpro software to create accompanying tables of results is also freely available (Figure 1) [**33**].

## Reporting quality

Reporting quality describes how well a piece of scientific work is written- as an article published by a scientific journal. A key criterion is whether the study as reported would allow a reader to replicate the work. Poor quality of reporting is common [34, 35], which can make it difficult to assess the methodology [35]. Various approaches to improving the quality of scientific reporting have been proposed. Successful examples include the guidance offered by Cochrane for systematic review [**36**] and the Enhancing the QUAlity and Transparency Of health Research (EQUATOR) Network. The EQUATOR Network provides resources for authors, editors and educationalists to promote all aspects of improving reporting quality in health research (Figure 1) [37,38].

Reporting guidance is presented in the form of a checklist of recommendations specific for the study design. The EQUATOR website includes a tool to assist in the selection of the correct guideline [**39**], as it can sometimes be difficult for researchers to be confident which is the most appropriate. The first of these guidelines was the Consolidated Standards of Reporting Trials (CONSORT) Statement for RCTs [40]. CONSORT now comprises a 22-item checklist assessing each section of the research paper [**41**]. CONSORT recommends the use of a structured flow diagram: describing numbers of participants at enrolment, allocation, follow-up and analysis [**42**]. Similar guidelines exist for the reporting of other study designs [**39**] and we encourage all authors to use these.

In reporting guidelines individual items are not weighted to indicate the quality the study report (and the completion of most or all items on the checklist is not necessarily an indicator of quality). For example, in Strengthening the Reporting of Observational Studies in Epidemiology (STROBE), one item is ‘indicate the study's design with a commonly used term in the title or abstract’ and another is ‘describe any efforts to assess potential sources of bias’ [**43**]. Clearly, a lack of consideration of bias will have a major impact on the results, whereas the title of the paper will not. Note that these are assessments of reporting quality, not an assessment of the methodology of the study itself.

## Are quality tools valuable?

Quality tools are widely available, but not universally accepted. Their use has improved the quality of reporting of studies [44, 45] with greater improvements in journals which promote or require their use [46, 47] and they can be useful for peer reviewers. Possible arguments against their use include the over-standardisation of published material. It can also challenge researchers if they do not have all the required information

Another limitation for some tools is their lack of external validation. Tool authors must seek external usage and testing of their tool to fully understand how it is applied by non-specialists. In particular identifying questions which may be open to interpretation can allow guidance notes to be provided.

## How and when to use the tools

### Critical appraisal for journal clubs, peer review or clinical use

For those delivering teaching about the principles of methodological quality assessment or running local journal clubs, there are plenty of resources available. ‘How to Read a Paper’ [**48**] is a short and accessible read and the Critical Appraisal Skills Programme Checklists [**49**] can be helpful for facilitating group sessions. Their 10–12 question checklists cover each of the key study designs. These are typically divided into three sections with prompts beside each question to help understanding [**49**]. They are largely designed to familiarise users with study designs and help them evaluate the relevance of the paper to their practice as they contain several subjective elements which may not lend themselves to incorporation in a formal quality assessment. Alternatively, the components of other methodological quality assessment tools can be used to facilitate discussion.

### Research study design

If you are at the beginning of a project, the tools can help identify factors to include in your design (e.g. who will assess your outcome and how can they be blinded to which participants have received the intervention). Similarly, if you are planning a systematic review, the reporting tools prompt you to find a second reviewer to improve the quality of your data. You also need to decide which tool to use to assess the methodological quality of the studies included in your review – be aware of their strengths and limitations.

### Research study reporting

Reporting quality checklists can help you structure the write up of your study, ensuring you present all recommended information. It is also important to be aware that many journals require that a completed checklist is submitted with your manuscript, indicating where you have addressed each point.

## Conclusions

Understanding and evaluating the evidence behind clinical practice is a valuable skill for any geriatrician. The formal tools which have been developed for assessment of methodological and reporting quality can be helpful when evaluating evidence or planning writing. Quality assessment is difficult and the most honest way to appraise studies is to look at the individual components of quality, rather than attempting a binary categorisation into high or low quality. These can then be presented in a transparent and understandable way, allowing you to determine whether the research is applicable to your clinical practice.

**Key points**

- Evaluating how well a study has been conducted is essential to determine if the findings are relevant to clinical practice.
- Making this assessment can be difficult depending on how well a study is reported.
- Guidelines are available to help assess the quality of a study's methods and to guide study reporting.
- These guidelines are useful for all clinicians wishing to critically appraise research findings for application in practice.

## Supplementary Material

Supplementary Data

Supplementary Data
