# Supplementary material for: Using quality assessment tools to critically appraise ageing research: a guide for clinicians
Source: Age Ageing. 2016 Dec 8;46(3):359–65. doi: 10.1093/ageing/afw223 (PMC5405751; doi:10.1093/ageing/afw223)
Supplement: Supplementary Data [file afw223_aa-16-0538-File001.docx]

**Supplementary Data**

**Appendix 1. Full Reference List**

1. Sackett DL. Rules of evidence and clinical recommendations on the use of antithrombotic agents. Chest. 1989 Feb;95(2 Suppl):2s-4s.

2. Burns A, Rossor M, Hecker J, Gauthier S, Petit H, Moller HJ, et al. The effects of donepezil in Alzheimer's disease - results from a multinational trial. Dement Geriatr Cogn Disord. 1999;10(3):237-44.

3. Cochrane Methods Bias. Assessing Risk of Bias in Included Studies. 2015 [cited 2015 8th October]; Available from: <http://bmg.cochrane.org/assessing-risk-bias-included-studies>.

4. Higgins JPT, Altman DG, Gøtzsche PC, Jüni P, Moher D, Oxman AD, et al. The Cochrane Collaboration’s tool for assessing risk of bias in randomised trials. BMJ. 2011;343:d5928.

5. Logan P, Armstrong S, Avery T, Barer D, Barton G, Darby J, et al. Rehabiliation aimed at improving outdoor mobility for people after stroke: a multicentre randomised controlled study (the Getting out of the House Study). Health Technology Assessment. 2014;18(29).

6. Habicht DW, Witham MD, McMurdo ME. The under-representation of older people in clinical trials: barriers and potential solutions. The journal of nutrition, health & aging. 2008 Mar;12(3):194-6.

7. Cochrane Informatics & Knowledge Management Department. Review Manager (RevMan) 5.3. 2014 [cited 2015 20th August]; Available from: <http://tech.cochrane.org/revman>.

8. Jadad AR, Moore RA, Carroll D, Jenkinson C, Reynolds DJ, Gavaghan DJ, et al. Assessing the quality of reports of randomized clinical trials: is blinding necessary? Control Clin Trials. 1996;17(1):1-12.

9. Clark HD, Wells GA, Huet C, McAlister FA, Salmi LR, Fergusson D, et al. Assessing the quality of randomized trials: reliability of the Jadad scale. Control Clin Trials. 1999;20(5):448-52.

10. Juni P, Witschi A, Bloch R, Egger M. The hazards of scoring the quality of clinical trials for meta-analysis. JAMA. 1999;282(11):1054-60.

11. Reeves B, Deeks J, Higgins J, Wells G. Chapter 13: Including non-randomized studies. In: Higgins J, Green S, editors. Cochrane Handbook for Systematic Reviews of Interventions: The Cochrane Collaboration; 2008.

12. Deeks J, Dinnes J, D'Amico R, Sowden A, Sakarovitch C, Song F, et al. Evaluating non-randomised intervention studies. Health Technology Assessment. 2003;7(27).

13. Sanderson S, Tatt I, Higgins J. Tools for assessing quality and susceptibility to bias in observational studies in epidemiology: a systematic review and annotated bibliography. International Journal of Epidemiology. 2007;36:666-76.

14. Inouye SK, Bogardus ST, Jr., Charpentier PA, Leo-Summers L, Acampora D, Holford TR, et al. A multicomponent intervention to prevent delirium in hospitalized older patients. The New England Journal of Medicine. 1999;340(9):669-76.

15. Kim S, Park J, Lee Y, Seo H-J, Sheen S-S, Hahn S, et al. Testing a tool for assessing the risk of bias for nonrandomized studies showed moderate reliability and promising validity. Journal of Clinical Epidemiology. 2013;66:408-14.

16. Greenhalgh T. How to read a paper. Getting your bearings (deciding what the paper is about). BMJ 1997 Jul 26;315(7102):243-6.

17. Davis DH, Barnes LE, Stephan BC, MacLullich AM, Meagher D, Copeland J, et al. The descriptive epidemiology of delirium symptoms in a large population-based cohort study: results from the Medical Research Council Cognitive Function and Ageing Study (MRC CFAS). BMC Geriatr. 2014;14:87.

18. Wells G, Shea B, O'Connell D, Peterson J, Welch V, Losos M, et al. The Newcastle-Ottawa Scale (NOS) for assessing the quality if nonrandomised studies in meta-analyses. 2014 [cited 2015 5th August]; Available from: <http://www.ohri.ca/programs/clinical_epidemiology/oxford.asp>.

19. Hartling L, Milne A, Hamm M, Vandermeer B, Ansari M, Tsertsvadze A, et al. Testing the Newcastle Ottawa Scale showed low reliability between individual reviewers. Journal of Clinical Epidemiology. 2013;66:982-93.

20. Stang A. Critical evaluation of the Newcastle-Ottawa scale for the assessment of the quality of nonrandomized studies in meta-analyses. European Journal of Epidemiology. 2010;25(9):603-5.

21. The Cochrane Collaboration. Cochrane Handbook for Systematic Reviews of Diagnostic Test Accuracy Version 1.0.0 2013 [cited 2015 8th October]. Available from: <http://srdta.cochrane.org/>.

22. Davis D, Creavin S, Noel-Storr A, Quinn T, Smailagic N, Hyde C, et al. Neuropsychological tests for the diagnosis of Alzheimer's disease dementia and other dementias: a generic protocol for cross-sectional and delayed-verification studies. Cochrane Database of Systematic Reviews. 2013(3).

23. Larner AJ. AD8 Informant Questionnaire for Cognitive Impairment: Pragmatic Diagnostic Test Accuracy Study. Journal of Geriatric Psychiatry and Neurology. 2015;28(3):198-202.

24. Whiting PF, Rutjes AWS, Westwood ME, Mallett S, Deeks JJ, Reitsma JB, et al. QUADAS-2: A Revised Tool for the Quality Assessment of Diagnostic Accuracy Studies. Annals of Internal Medicine. 2011;155(8):529-36.

25. Harrison J, Fearon P, Noel-Storr A, McShane R, Stott D, Quinn T. Informant Questionnaire on Cognitive Decline in the Elderly (IQCODE) for the diagnosis of dementia within a secondary care setting. Cochrane Database of Systematic Reviews. 2015(3).

26. Reilly S, Miranda-Castillo C, Malouf R, Hoe J, Toot S, Challis D, et al. Case management approaches to home support for people with dementia. The Cochrane database of systematic reviews. 2015;1:Cd008345.

27. Shea BJ, Grimshaw JM, Wells GA, Boers M, Andersson N, Hamel C, et al. Development of AMSTAR: a measurement tool to assess the methodological quality of systematic reviews. BMC Med Res Methodol. 2007;7:10.

28. Shea BJ, Hamel C, Wells GA, Bouter LM, Kristjansson E, Grimshaw J, et al. AMSTAR is a reliable and valid measurement tool to assess the methodological quality of systematic reviews. Journal of Clinical Epidemiology. 2009;62(10):1013-20.

29. Shea BJ, Bouter LM, Peterson J, Boers M, Andersson N, Ortiz Z, et al. External Validation of a Measurement Tool to Assess Systematic Reviews (AMSTAR). PLoS One. 2007;2(12):e1350.

30. Kang D, Wu Y, Hu D, Hong Q, Wang J, Zhang X. Reliability and External Validity of AMSTAR in Assessing Quality of TCM Systematic Reviews. Evidence-Based Complementary and Alternative Medicine. 2012;2012:732195.

31. GRADE Working Group. Grading quality of evidence and strength of recommendations. BMJ. 2004;328(7454):1490.

32. Guyatt GH, Oxman AD, Vist GE, Kunz R, Falck-Ytter Y, Alonso-Coello P, et al. GRADE: an emerging consensus on rating quality of evidence and strength of recommendations. BMJ. 2008;336(7650):924-6.

33. McMaster University and Evidence Prime Inc. GRADEpro Guideline Development Tool. 2015 [cited 2015 20th August]; Available from: <http://www.guidelinedevelopment.org/>.

34. Pocock S, Collier T, Dandreo K, de Stavola B, Goldman M, Kalish L, et al. Issues in the reporting of epidemiological studies: a survey of recent practice. BMJ. 2004;329(7471):883.

35. Chan A-W, Altman DG. Epidemiology and reporting of randomised trials published in PubMed journals. The Lancet. 2005;365(9465):1159-62.

36. Cochrane Handbook for Systematic Reviews of Interventions Version 5.1. [updated March 2011]. The Cochrane Collaboration 2011 [cited 2016 30th May]. Available from: <http://handbook.cochrane.org>.

37. Equator Network. Enhancing the QUAlity and Transparency Of health Research. [cited 2015 17th August]; Available from: <http://www.equator-network.org>.

38. Simera I, Altman D, Moher D, Schulz K, Hoey J. Guidelines for Reporting Health Research: The EQUATOR Network's Survey of Guideline Authors. PLoS Med. 2008;5(6):e139.

39. Equator Network. Selecting the appropriate reporting guideline for your article. Available from: <http://www.equator-network.org/toolkits/teachers/spotting-study-design-and-selecting-appropriate-reporting-guideline/>.

40. Begg C, Cho M, Eastwood S, Horton R, Moher D, Olkin I, et al. Improving the quality of reporting of randomized controlled trials. The CONSORT statement. JAMA. 1996;276(8):637-9.

41. Schulz K, Altman D, Moher D. CONSORT 2010 Statement: updated guidelines for reporting parallel group randomised trials. BMJ. 2010;152(11):726-32.

42. Moher D, Schulz K, Altman D, for the CONSORT Group. The CONSORT Statement: revised recommendations for improving the quality of reports of parallel-group randomised trials. The Lancet. 2001;357:1191-94.

43. von Elm E, Altman D, Egger M, Pocock S, Gotzsche P, Vandenbroucke J, et al. The Strengthening the Reporting of Observational Studies in Epidemiology (STROBE) statement: guidelines for reporting observational studies. Journal of Clinical Epidemiology. 2008;61:344-9.

44. Kane R, Wang J, Garrard J. Reporting in randomized clinical trials improved after adoption of the CONSORT statement. Journal of Clinical Epidemiology. 2007;60(3):241-9.

45. Korevaar D, van Enst W, Spijker R, Bossuyt P, Hooft L. Reporting quality of diagnostic accuracy studies: a systematic review and meta-analysis of investigations on adherence to STARD. Evidence Based Medicine. 2014;19(2):47-54.

46. Devereaux P, Manns B, Ghali W, Quan H, Guyatt G. The reporting of methodological factors in randomized controlled trials and the association with a journal policy to promote adherence to the Consolidated Standards of Reporting Trials (CONSORT) checklist. Controlled Clinical Trials. 2002;23:380-88.

47. Smidt N, Rutjes A, van der Windt D, Ostelo R, Bossuyt P, Reitsma J, et al. The quality of diagnostic accuracy studies since the STARD statement. Has it improved? Neurology. 2006;67:792-7.

48. Greenhalgh T. How to Read a Paper. 5th ed. UK: Wiley Blackwell & BMJ Books,; 2014.

49. Critical Appraisal Skills Programme. CASP Checklists. 2013 [cited 2015 18th September ]; Available from: <http://www.casp-uk.net/#!checklists/cb36>.

**Appendix 2: Example of Cochrane Risk of Bias Table for a randomised controlled clinical trial**


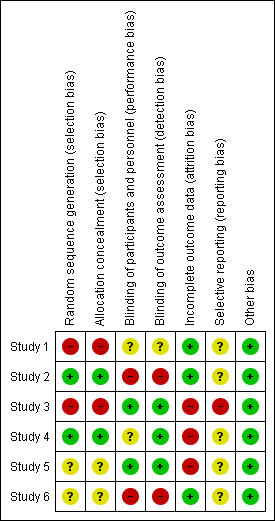


Table 1: Methodological considerations pertinent to research in the older adult population

| Most quality assessment or risk of bias tools are generic, however there are certain considerations that are more important in older adult research than in other research areas. We present a summary of some of these aspects to inform the design and interpretation of quality assessment. For a more detailed discussion of these points we would recommend the excellent reviews by McMurdo *et al.*[1] and Witham & McMurdo[2].:  ARE THE RESEARCH QUESTIONS AND OUTCOME MEASURES RELEVANT TO OLDER ADULTS?: Research is of greatest value if it considers the priorities of patients, carers and service users. For older adults, outcomes such as quality of life and maintaining independence may be more important that traditional outcomes such as mortality. The James Lind Alliance have a program of work designed to describe the research priorities of patients and other stake holders.[3] Data should be able to be collected from those with physical, sensory and cognitive impairments.  HAVE OLDER ADULTS BEEN INVOLVED IN STUDY PLANNING, CONDUCT AND INTERPRETATION?: Involving stakeholders in all aspects of the research process has many benefits (ensuring study materials are appropriately worded, assessing treatment and assessment burden). Many study funders mandate patient involvement but how are representative views sought for such a diverse group? This can be challenging in some groups, such as care home residents or those with advanced dementia.[4]  IS THE STUDY DESIGNED FOR ATTRITION OF OLDER ADULTS?: Loss to follow-up and study “drop-outs” can impact on the validity of study results and often feature in quality assessment tools; however in older adult research a higher attrition rate is expected. Attrition of older adults is not necessarily a marker of a poor quality study but analysis methods and sample size calculations should be appropriately adjusted for this.  ARE RECRUITMENT PROCEDURES APPROPRIATE FOR OLDER ADULTS?: Where have recruitment and assessments have taken place, and have attempts been made to reach those who cannot come to a hospital clinic? The Newcastle 85+ study found more than half of their pilot study group were unable or unwilling to attend hospital, so they introduced recruitment and assessment in the individual’s current residence.[5]  DOES THE STUDY ATTEMPT TO INCLUDE THOSE WHO LACK CAPACITY?: Many of the important research areas in geriatric medicine are likely to affect those who lack capacity to consent to participate. Representative research must therefore include mechanisms to include this population using appropriate ethical and legal processes. The PiTSTOP study, conducted in English care homes, established a recruitment procedure which allowed for consent to be provided by a relative or professional caregiver where the individual lacked capacity to consent, in accordance with the requirements of the Mental Capacity Act.[6]  ARE ALL OLDER ADULTS THAT MAY BENEFIT FROM THE INTERVENTION INCLUDED IN THE STUDY?: The generalisability of findings and adoption into practice depends on the original representativeness of those included. Ensuring older adults are not excluded on grounds of age, co-morbidity or frailty either directly, through exclusion in study criteria or indirectly, through study procedures is a key priority for researchers. |
| --- |

Table 3: Comparing the components of tools for observational study designs

|  | **Newcastle-Ottawa[7]** | **Newcastle-Ottawa[7]** | **Downs & Black[8]** | **RoBANS[9]** |
| --- | --- | --- | --- | --- |
| **Study designs** | Case-control studies | Cohort studies | Randomised and non-randomised  (including cohort and case-control) | Non-randomised |
| **Purpose** | Methodological quality | Methodological quality | Reporting and methodological quality | Risk of bias |
|  | **Included Domains/Questions** | | | |
|  | **Selection**   1. Is the case definition adequate? 2. Representativeness of the cases 3. Selection of controls 4. Definition of controls   **Comparability**   1. Comparability of cases and controls on the basis of the design or the analysis   **Exposure**   1. Ascertainment of exposure 2. Same method of ascertainment for cases and controls? 3. Non-response rate | **Selection**   1. Representativeness of the exposed cohort 2. Selection of the non-exposed cohort 3. Ascertainment of exposure 4. Demonstration that the outcome of interest was not present at start of the study   **Comparability**   1. Comparability of cohorts on the basis of the design or the analysis   **Outcome**   1. Ascertainment of outcome 2. Was follow-up long enough for outcomes to occur? 3. Adequacy of follow-up of cohorts | 1. Reporting: 10 questions 2. External validity: 3 questions 3. Internal validity – bias: 7 questions 4. Internal validity – confounding (selection bias): 6 questions 5. Power: 1 question | 1. Selection of participants (selection bias) 2. Confounding variables (selection bias) 3. Intervention (exposure) measurement (performance bias) 4. Blinding of outcome assessment (detection bias) 5. Incomplete outcome data (attrition bias) 6. Selective outcome reporting (reporting bias) |

Reference List

1. McMurdo ME, Roberts H, Parker S, Wyatt N, May H, Goodman C, et al. Improving recruitment of older people to research through good practice. Age Ageing. 2011 Nov;40(6):659-65.

2. Witham M, McMurdo M. How to Get Older People Included in Clinical Studies. Drugs & Aging. 2007;24(3):187-96.

3. Kelly S, Lafortune L, Hart N, Cowan K, Fenton M, Brayne C. Dementia priority setting partnership with the James Lind Alliance: using patient and public involvement and the evidence base to inform the research agenda. Age & Ageing. 2015;44(6):985-93.

4. Backhouse T, Kenkmann A, Lane K, Penhale B, Poland F, Killett A. Older care-home residents as collaborators or advisors in research: a systematic review. Age Ageing. 2016 May;45(3):337-45.

5. Collerton J, Barrass K, Bond J, Eccles M, Jagger C, James O, et al. The Newcastle 85+ study: biological, clinical and psychosocial factors associated with healthy ageing: study protocol. BMC Geriatr. 2007;7:14.

6. Siddiqi N, Cheater F, Collinson M, Farrin A, Forster A, George D, et al. The PiTSTOP study: a feasibility cluster randomized trial of delirium prevention in care homes for older people. Age Ageing. 2016 May 20.

7. Wells G, Shea B, O'Connell D, Peterson J, Welch V, Losos M, et al. The Newcastle-Ottawa Scale (NOS) for assessing the quality if nonrandomised studies in meta-analyses. 2014 [cited 2015 5th August]; Available from: <http://www.ohri.ca/programs/clinical_epidemiology/oxford.asp>.

8. Downs S, Black N. The feasibility of creating a checklist for the assessment of the methodological quality both of randomised and non-randomised studies of health care interventions. Journal of Epidemiology and Community Health. 1998;52:377-84.

9. Kim S, Park J, Lee Y, Seo H-J, Sheen S-S, Hahn S, et al. Testing a tool for assessing the risk of bias for nonrandomized studies showed moderate reliability and promising validity. Journal of Clinical Epidemiology. 2013;66:408-14.
