# Supplementary material for: Using quality assessment tools to critically appraise ageing research: a guide for clinicians
Source: Age Ageing. 2016 Dec 8;46(3):359–65. doi: 10.1093/ageing/afw223 (PMC5405751; doi:10.1093/ageing/afw223)
Supplement: Supplementary Data [file afw223_SupplementaryFile1.docx]

Supplementary File

Figure 1: Example of Cochrane Risk of Bias Table for a randomised controlled clinical trial


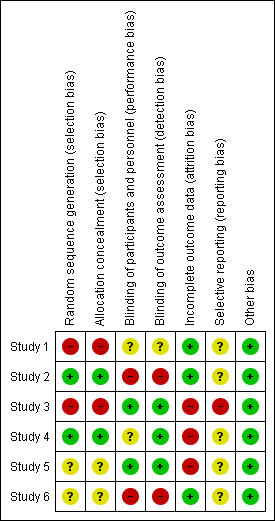


Table 1: Methodological considerations pertinent to research in the older adult population

| Most quality assessment or risk of bias tools are generic, however there are certain considerations that are more important in older adult research than in other research areas. We present a summary of some of these aspects to inform the design and interpretation of quality assessment. For a more detailed discussion of these points we would recommend the excellent reviews by McMurdo *et al.*[1] and Witham & McMurdo[2].:  ARE THE RESEARCH QUESTIONS AND OUTCOME MEASURES RELEVANT TO OLDER ADULTS?: Research is of greatest value if it considers the priorities of patients, carers and service users. For older adults, outcomes such as quality of life and maintaining independence may be more important that traditional outcomes such as mortality. The James Lind Alliance have a program of work designed to describe the research priorities of patients and other stake holders.[3] Data should be able to be collected from those with physical, sensory and cognitive impairments.  HAVE OLDER ADULTS BEEN INVOLVED IN STUDY PLANNING, CONDUCT AND INTERPRETATION?: Involving stakeholders in all aspects of the research process has many benefits (ensuring study materials are appropriately worded, assessing treatment and assessment burden). Many study funders mandate patient involvement but how are representative views sought for such a diverse group? This can be challenging in some groups, such as care home residents or those with advanced dementia.[4]  IS THE STUDY DESIGNED FOR ATTRITION OF OLDER ADULTS?: Loss to follow-up and study “drop-outs” can impact on the validity of study results and often feature in quality assessment tools; however in older adult research a higher attrition rate is expected. Attrition of older adults is not necessarily a marker of a poor quality study but analysis methods and sample size calculations should be appropriately adjusted for this.  ARE RECRUITMENT PROCEDURES APPROPRIATE FOR OLDER ADULTS?: Where have recruitment and assessments have taken place, and have attempts been made to reach those who cannot come to a hospital clinic? The Newcastle 85+ study found more than half of their pilot study group were unable or unwilling to attend hospital, so they introduced recruitment and assessment in the individual’s current residence.[5]  DOES THE STUDY ATTEMPT TO INCLUDE THOSE WHO LACK CAPACITY?: Many of the important research areas in geriatric medicine are likely to affect those who lack capacity to consent to participate. Representative research must therefore include mechanisms to include this population using appropriate ethical and legal processes. The PiTSTOP study, conducted in English care homes, established a recruitment procedure which allowed for consent to be provided by a relative or professional caregiver where the individual lacked capacity to consent, in accordance with the requirements of the Mental Capacity Act.[6]  ARE ALL OLDER ADULTS THAT MAY BENEFIT FROM THE INTERVENTION INCLUDED IN THE STUDY?: The generalisability of findings and adoption into practice depends on the original representativeness of those included. Ensuring older adults are not excluded on grounds of age, co-morbidity or frailty either directly, through exclusion in study criteria or indirectly, through study procedures is a key priority for researchers. |
| --- |

Table 3: Comparing the components of tools for observational study designs

|  | **Newcastle-Ottawa[7]** | **Newcastle-Ottawa[7]** | **Downs & Black[8]** | **RoBANS[9]** |
| --- | --- | --- | --- | --- |
| **Study designs** | Case-control studies | Cohort studies | Randomised and non-randomised  (including cohort and case-control) | Non-randomised |
| **Purpose** | Methodological quality | Methodological quality | Reporting and methodological quality | Risk of bias |
|  | **Included Domains/Questions** | | | |
|  | **Selection**   1. Is the case definition adequate? 2. Representativeness of the cases 3. Selection of controls 4. Definition of controls   **Comparability**   1. Comparability of cases and controls on the basis of the design or the analysis   **Exposure**   1. Ascertainment of exposure 2. Same method of ascertainment for cases and controls? 3. Non-response rate | **Selection**   1. Representativeness of the exposed cohort 2. Selection of the non-exposed cohort 3. Ascertainment of exposure 4. Demonstration that the outcome of interest was not present at start of the study   **Comparability**   1. Comparability of cohorts on the basis of the design or the analysis   **Outcome**   1. Ascertainment of outcome 2. Was follow-up long enough for outcomes to occur? 3. Adequacy of follow-up of cohorts | 1. Reporting: 10 questions 2. External validity: 3 questions 3. Internal validity – bias: 7 questions 4. Internal validity – confounding (selection bias): 6 questions 5. Power: 1 question | 1. Selection of participants (selection bias) 2. Confounding variables (selection bias) 3. Intervention (exposure) measurement (performance bias) 4. Blinding of outcome assessment (detection bias) 5. Incomplete outcome data (attrition bias) 6. Selective outcome reporting (reporting bias) |

Reference List

1. McMurdo ME, Roberts H, Parker S, Wyatt N, May H, Goodman C, et al. Improving recruitment of older people to research through good practice. Age Ageing. 2011 Nov;40(6):659-65.

2. Witham M, McMurdo M. How to Get Older People Included in Clinical Studies. Drugs & Aging. 2007;24(3):187-96.

3. Kelly S, Lafortune L, Hart N, Cowan K, Fenton M, Brayne C. Dementia priority setting partnership with the James Lind Alliance: using patient and public involvement and the evidence base to inform the research agenda. Age & Ageing. 2015;44(6):985-93.

4. Backhouse T, Kenkmann A, Lane K, Penhale B, Poland F, Killett A. Older care-home residents as collaborators or advisors in research: a systematic review. Age Ageing. 2016 May;45(3):337-45.

5. Collerton J, Barrass K, Bond J, Eccles M, Jagger C, James O, et al. The Newcastle 85+ study: biological, clinical and psychosocial factors associated with healthy ageing: study protocol. BMC Geriatr. 2007;7:14.

6. Siddiqi N, Cheater F, Collinson M, Farrin A, Forster A, George D, et al. The PiTSTOP study: a feasibility cluster randomized trial of delirium prevention in care homes for older people. Age Ageing. 2016 May 20.

7. Wells G, Shea B, O'Connell D, Peterson J, Welch V, Losos M, et al. The Newcastle-Ottawa Scale (NOS) for assessing the quality if nonrandomised studies in meta-analyses. 2014 [cited 2015 5th August]; Available from: <http://www.ohri.ca/programs/clinical_epidemiology/oxford.asp>.

8. Downs S, Black N. The feasibility of creating a checklist for the assessment of the methodological quality both of randomised and non-randomised studies of health care interventions. Journal of Epidemiology and Community Health. 1998;52:377-84.

9. Kim S, Park J, Lee Y, Seo H-J, Sheen S-S, Hahn S, et al. Testing a tool for assessing the risk of bias for nonrandomized studies showed moderate reliability and promising validity. Journal of Clinical Epidemiology. 2013;66:408-14.
